# MR imaging of proton beam‐induced oxygen depletion

**DOI:** 10.1002/mp.17622

**Published:** 2025-01-28

**Authors:** Juliane Schieferecke, Aswin Hoffmann, Jörg Pawelke

**Affiliations:** ^1^ OncoRay ‐ National Center for Radiation Research in Oncology Faculty of Medicine and University Hospital Carl Gustav Carus Technische Universität Dresden Helmholtz‐Zentrum Dresden‐Rossendorf Dresden Germany; ^2^ Helmholtz‐Zentrum Dresden‐Rossendorf Institute of Radiooncology – OncoRay Dresden Germany; ^3^ Department of Radiotherapy and Radiation Oncology Faculty of Medicine and University Hospital Carl Gustav Carus Technische Universität Dresden Germany

**Keywords:** MRI‐based proton beam visualization, MR‐integrated proton therapy, oxygen depletion

## Abstract

**Background:**

Previous studies have shown that in‐beam magnetic resonance imaging (MRI) can be used to visualize a proton beam during the irradiation of liquid‐filled phantoms. The beam energy‐ and current‐dependent local image contrast observed in water was identified to be predominantly caused by beam‐induced buoyant convection and associated flow effects. Besides this flow dependency, the MR signal change was found to be characterized by a change in the $T_{1}$ relaxation time of water, hinting at a radiochemical contribution, which was hypothesized to lie in oxygen depletion‐evoked $T_{1}$ relaxation time lengthening. The elucidation of the underlying contrast mechanism is required to enable the further assessment of the application potential of MRI‐based proton beam visualization in tissue.

**Purpose:**

The underlying radiochemical cause of the observed local beam‐induced change in the $T_{1}$ relaxation time of water should be identified in beam visualization experiments testing the hypothesis of beam‐induced oxygen depletion‐evoked $T_{1}$ relaxation time lengthening.

**Methods:**

Combined irradiation and imaging experiments were performed using static proton pencil beam irradiation, background‐nulled inversion recovery (IR) MRI and a range of flow‐restricted phantoms differing in initial oxygen concentration and homogeneity. The similarity of the irradiation‐induced MRI contrast to the proton pencil beam dose distribution acquired on radiochromic film, the expected dose dependence and temporal stability, the TR dependence as well as the dependence on the initial oxygen concentration and the oxygen consumption rate were tested. Moreover, the feasibility of oxygen depletion‐based beam visualization in tissue‐mimicking phantoms was assessed. The levels of irradiation‐induced oxygen depletion and $T_{1}$ relaxation time lengthening were estimated based on the measured temperatures and initial oxygen concentrations of the phantoms, the experimentally determined inversion times required for phantom background signal nulling and dosimetric measurements.

**Results:**

The proton irradiation‐induced contrast in background‐nulled IR images of well oxygenated phantoms was found similar to the proton pencil beam dose distribution and showed the characteristics expected for oxygen depletion‐induced MRI contrast. No beam‐induced contrast was observed in the poorly oxygenated, inhomogeneous tissue‐mimicking phantoms.

**Conclusions:**

Proton beam‐induced radiochemical oxygen depletion can be visualized using $T_{1}$ relaxation time contrast‐based IR MRI and represents the first identified flow‐independent contrast mechanism in MRI‐based proton beam visualization in real‐time. Beam detection in tissue, however, will be complicated by the increased $T_{1}$ relaxation time inhomogeneity and the lowered levels of initial oxygen concentration compared to in liquids at atmospheric equilibrium and requires further assessment.

## 1 INTRODUCTION

The postulated dosimetric advantage of proton beam radiation therapy over conventional photon beam irradiation is based on its inverse depth‐dose profile, in which the dose maximum occurs distally from the dose plateau region at the end of the beam's energy‐dependent range in the so‐called Bragg peak. Because of the associated steeper dose gradients, proton therapy is highly sensitive to patient‐individual organ motion and deformation as the finite range of the proton beam in tissue is determined by the material composition along its path.^1^ In‐beam magnetic resonance imaging (MRI) does not only offer real‐time monitoring of these anatomical changes, which is expected to improve the targeting accuracy and precision of proton therapy for moving tumors in particular,^2^, ^3^ but may additionally enable the verification of dose delivery by means of direct, non‐invasive proton beam visualization as demonstrated in preceding proof‐of‐principle studies.^4^, ^5^

These initial studies in MRI‐based proton beam visualization were mainly conducted in liquid‐filled phantoms and showed local MR signal loss within the beam volume. The contrast generation was subsequently found to strongly depend on beam‐induced buoyant convection and the directional flow associated,^6^, ^7^ making its application in flow‐restricting tissue virtually impossible while being potentially useful for quality assurance (QA) applications in MR‐integrated proton therapy. However, in our magnitude signal‐based convection study, weak, hitherto unexplained local magnitude signal loss was observed in a flow‐restricted water phantom using a strongly $T_{1}$‐weighted time‐of‐flight angiography pulse sequence, excluding convection to have caused the signal change. Moreover, a $T_{1}$‐weighting dependence of the beam‐induced hypointense contrast was discovered.^7^ Both observations may be indicative of the presence of MRI‐detectable radiochemical changes in the irradiated material resulting in the local lengthening of the $T_{1}$ relaxation time, potentially representing an irradiation‐induced contrast mechanism that may enable beam visualization in flow‐restricted tissue.

Only recently, the feasibility of the MRI‐based visualization of photon beams in aqueous phantoms based on radiochemical oxygen depletion was demonstrated, with the irradiation resulting in $T_{1}$ relaxation time lengthening detectable using an inversion recovery (IR) spin echo pulse sequence on a 0.35 T hybrid MRI linear accelerator (MR‐linac).^8^ Since oxygen depletion has been shown to not only occur during photon, but also during proton beam irradiation of oxygenated material,^9^, ^10^ the hypothesis that radiochemical oxygen depletion may result in visible MR signal changes can equally be raised for proton beam visualization.

The purpose of the present study was therefore to experimentally assess this oxygen depletion hypothesis for MRI‐based proton beam visualization, to thereby possibly explain the previously inexplicable experimental findings and to investigate the method's potential for MRI‐based proton beam monitoring in tissue.

## 2 METHODS

### 2.1 Experimental setup

**FIGURE 1:**
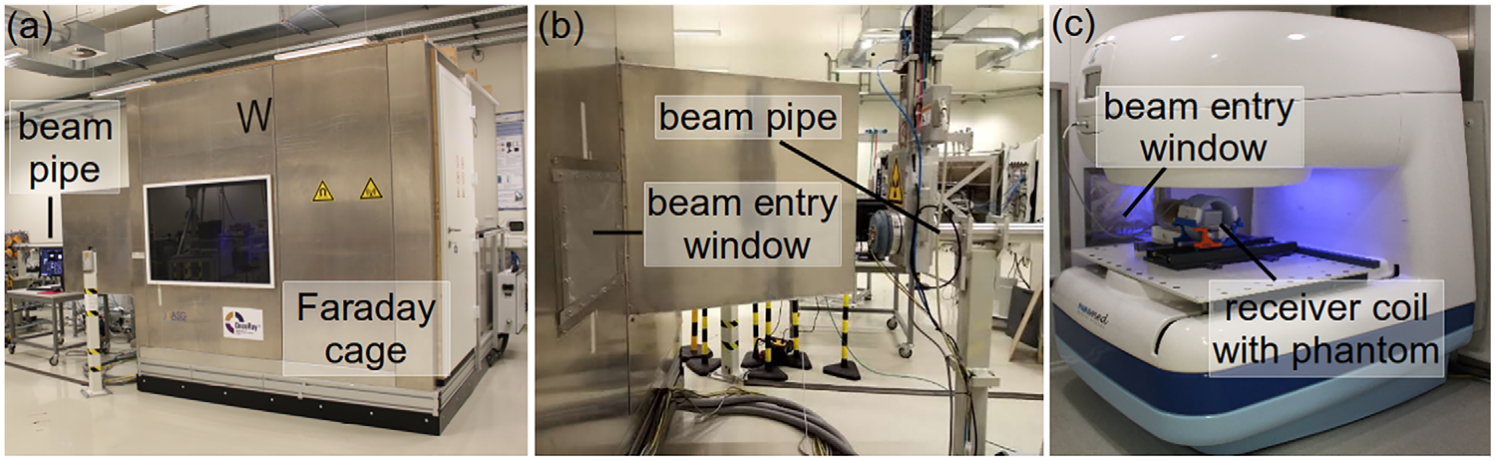
In‐beam MRI scanner positioned downstream of the horizontal proton research beamline. (a) The RF cabin hosts the scanner. (b) Closeup of the distal end of the beam pipe and the beam entry window of the RF cabin, taken from the opposite side. (c) The MRI scanner is located distally from the beam entry window inside the RF cabin. MRI, magnetic resonance imaging; RF, radiofrequency.

**FIGURE 2:**
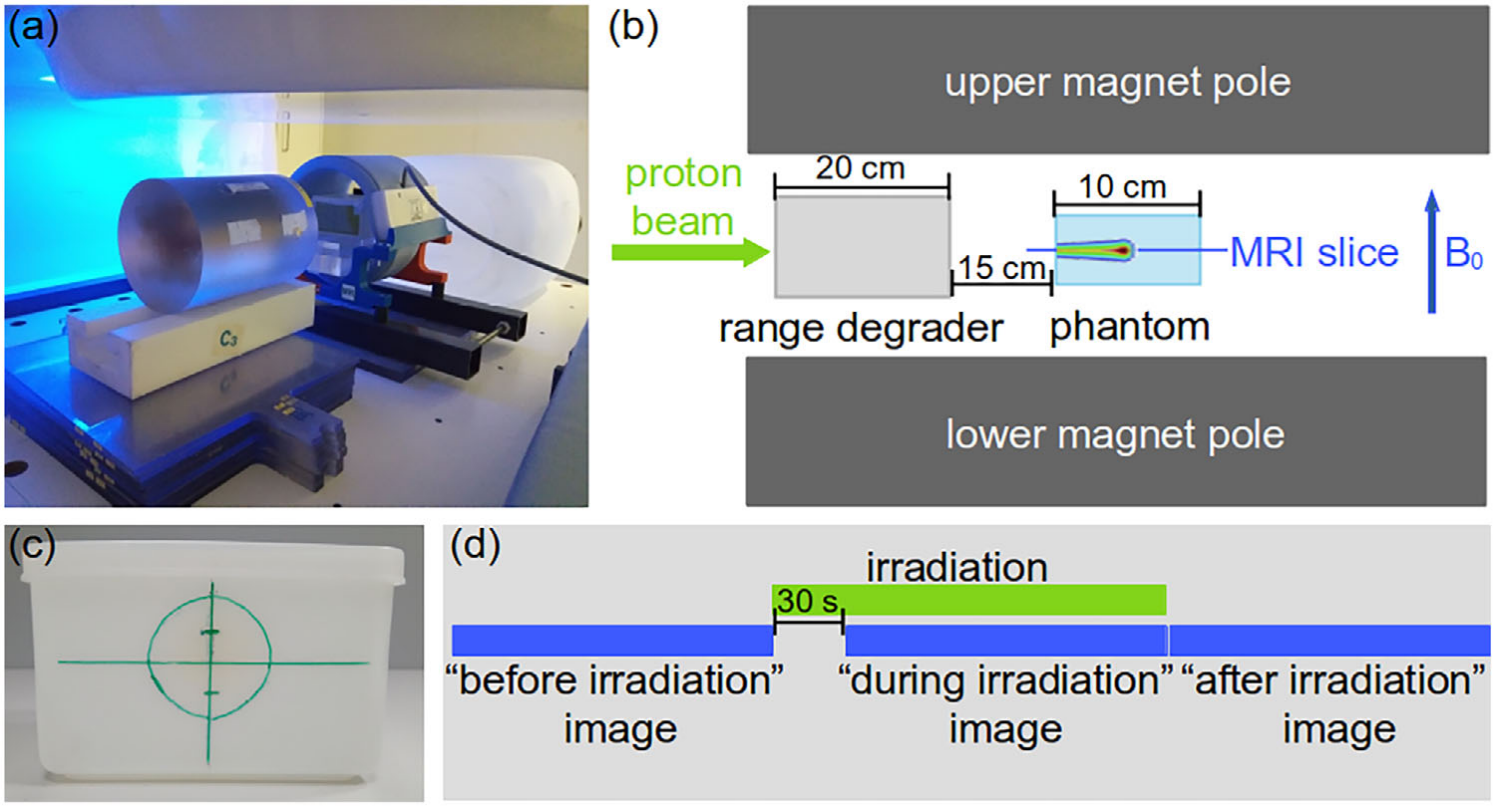
Experimental setup of the combined irradiation and imaging experiments. (a) Closeup of the range degrader positioned in front of the phantom placed in the receiver coil. (b) Schematic drawing of the setup within the RF cabin and imaging geometry. The single slice coronal MR images intersected the pencil beam volume horizontally and were acquired perpendicular to the vertical direction of the main magnetic field $B_{0}$. (c) Front view of the main imaging and dosimetry phantom container. (d) Timing of the beam visualization experiments with image acquisitions being performed before, during and immediately after irradiation. A 30 s pre‐irradiation interval precedes the period of simultaneous irradiation and imaging.

A mobile, radiofrequency (RF)‐shielded 0.32 T permanent magnet‐based in‐beam MRI scanner (MrJ3300, ASG Superconductors S.p.A., Genoa, Italy) was used for the combined irradiation and imaging experiments (Figure 1). Taking into account the horizontal deflection of the high‐energy proton beam by the vertically upwards oriented static magnetic field of the scanner, it was positioned in front of the horizontal research beamline for stationary pencil beam delivery at the University Proton Therapy Dresden facility, which is fed by an isochronous cyclotron (C230, Ion Beam Applications SA, Louvain‐la‐Neuve, Belgium). After passing through the 20 μm thick aluminium beam entry window of the RF cabin, the proton beam subsequently traversed a 20 cm thick polymethyl methacrylate (PMMA) range degrader to finally be stopped within the phantom positioned inside a knee receiver coil (Figure 2a,b).

### 2.2 Phantoms

Cuboid plastic containers with nominal outer dimensions of 100 × 100 × 65 mm^3^ (Figure 2c) were used to build floral foam‐ and agarose‐based phantoms. To build the “floral foam” phantom, one such container was filled with flow‐restricting phenolic resin‐based wet floral foam (GLOREX AG, Füllinsdorf, Switzerland), which is conventionally used to keep floral arrangements wet, and soaked with tap water. This phantom served to connect the oxygen depletion experiments to a preceding beam visualization study, which found unexplained weak motion‐independent signal changes in a similar foam phantom.^7^ Moreover, because of the better chemical characterization of agarose compared to floral foam, three different 1%w/v agarose gel phantoms (Agarose A9539, Sigma Life Science, Burlington, MA, USA) were prepared using distilled water. These phantoms served to test the influence of the initial oxygen concentration and the modification of the oxygen consumption rate on beam‐induced image contrast. To build the pure “agarose” phantom, the agarose preparation was heated until melting and then poured into the container. For the “agarose + oxygen” phantom, a double concentrated agarose solution of half the final phantom volume was prepared by heating while oxygen gas was bubbled through an equal volume of distilled water at room temperature for approximately 20 min. Subsequently, this agarose solution and the oxygen‐gassed water were mixed and gassed for another 3 min before being poured into the phantom container. For the “agarose + coumarin” phantom, in which the oxygen consumption rate is enhanced by coumarin undergoing radiochemical hydroxylation,^8^ a 10 mM coumarin solution (Coumarin ≥ 99%, Sigma Aldrich Chemie GmbH, Steinheim, Germany) was first prepared in distilled water by heating and left to cool down and reoxygenate under atmospheric conditions for 2 days. The phantom was then prepared by melting agarose in this solution. In addition to these phantoms, a piece of scalded Lyoner sausage and pieces of fresh pork liver tightly packed in a smaller‐sized cuboid plastic container served as two tissue‐mimicking phantoms. All phantoms were first placed into the RF cabin to equilibrate to the ambient temperature of about $28^{\circ }C$ ± $1^{\circ }C$ before they were centrally positioned in the receiver coil placed at the MRI isocentre for the beam visualization experiments.

### 2.3 MR imaging and quantitative image analysis

A 2D IR pulse sequence with fast spin echo readout was used to acquire single slice images intersecting the beam volume horizontally (Figure 2b). The corresponding signal equation^12^

(1) $$M_{z}=M_{0}\left[1-2e^{-TI/T_{1}}+e^{-\left(TR-TE_{last}\right)/T_{1}}\right]$$

describes the longitudinal magnetization $M_{z}$, with the equilibrium magnetization $M_{0}$, the inversion time TI, the longitudinal relaxation time $T_{1}$, the repetition time TR and the echo time of the last echo $TE_{last}$. ${\text{TE}}_{last}$ can be calculated from the echo train length, ETL, and the echo spacing, ESP, using

(2) $${\text{TE}}_{\mathrm{last}}=\mathrm{ETL}\times \mathrm{ESP}.$$

The correct vertical slice positioning was verified by acquisition of time‐of‐flight angiography beam visualization images during proton beam irradiation of an equally sized water‐filled phantom.^7^ The following sequence parameters were used for all experiments: TE = 30 ms, ESP = 15 ms, ETL = 3, number of excitations = 1, field‐of‐view = 180 × 180 mm^2^, samples = 64, encodings = 64, slice thickness = 10 mm. TRs of either 2000 or 5000 ms with corresponding total scan durations of 56 and 140 s, respectively, were used. The TIs used for background signal nulling were determined experimentally and ranged from 110 to 1460 ms, depending on TR and phantom material. Phase encoding was applied perpendicular to the beam's central axis because this orientation proved favorable in terms of imaging artefacts in our previous studies using the 0.22 T in‐beam MRI scanner.^5^, ^6^, ^7^ Magnitude signal‐reconstructed DICOM images with an in‐plane resolution of 2.02 × 2.02 mm^2^ were used for analysis and display. Beam‐induced contrast was assessed by averaging of the signal intensity in regions of interest within the beam and phantom background volume, respectively, and subsequent quotient formation.

### 2.4 Determination of the oxygen concentrations of the unirradiated phantoms

An optical bare fibre sensor‐based oxygen monitor (OxyLite, Oxford Optronix Ltd., Abingdon, UK) capable of quantifying the partial pressure of oxygen in fluids and tissues by molecular oxygen‐induced quenching of fluorescent light in a temperature‐independent mode was used for oxygen concentration measurement in the phantoms prior to irradiation. No optical probe‐based measurement of the oxygen concentrations during or after irradiation was performed in the present study because of geometrical restrictions within the MR receiver coil and the high levels of phantom activation, that ruled out the prompt removal of the phantom for measurement. The sensor's measurement range was limited to oxygen concentrations of 0 to 326 μM. Using the appropriate procedure described by the vendor depending on the sample type, measurements were obtained at at least two sensor insertion sites per phantom. After the stabilization of the displayed quasi‐continuous measurement values, single readings were obtained per measurement site and averaged to yield the oxygen concentration assumed to prevail throughout the phantom. Since the initial oxygen concentration of the “agarose + oxygen” phantom exceeded the sensor's measurement range of 0 to 326 μM, it was estimated based on the oxygen concentrations measured in the “agarose” and “agarose + coumarin” phantoms. The equation

(3) $$TI=T_{1}\left[\ln2-\ln\left(1+e^{-\left(TR-TE_{last}\right)/T_{1})}\right)\right]$$

was used to first iteratively approximate the $T_{1}$ relaxation times of all three agarose‐based phantoms from the respective TI value required for signal nulling in the respective phantom at $28^{\circ }C$ phantom temperature.^12^ These signal nulling TI values were individually determined by variation of the TI in subsequent image acquisitions in progressively smaller steps of down to 1 ms around the final value and intermittent calculation of the signal‐to‐noise ratio, which was minimized in this process. The differences in the oxygen concentration of the “agarose + oxygen” phantom relative to those of the “agarose” and “agarose + coumarin” phantoms, $\Delta c_{Ox,phantoms}$, were subsequently calculated based on the $T_{1}$ estimate obtained for the “agarose + oxygen” phantom, $T_{1_{phantom}}$, the $T_{1}$ estimate for the “agarose” or “agarose + coumarin” reference phantom, respectively, $T_{1_{reference}}$, and the phantom temperature and magnetic field‐dependent relaxivity value of oxygen, $r1_{Ox}$, using the equation^8^

(4) $$\Delta c_{Ox,phantoms}=\left(1/T_{1_{phantom}}-1/T_{1_{reference}}\right)/r1_{Ox}.$$

The initial oxygen concentration of the “agarose + oxygen” phantom was finally obtained by adding the respective estimated difference in the oxygen concentration to the respective initial oxygen concentration measured in the unirradiated “agarose” and “agarose + coumarin” phantoms, respectively, and subsequent averaging of both resulting oxygen concentration values.

### 2.5 Dosimetry

**FIGURE 3:**
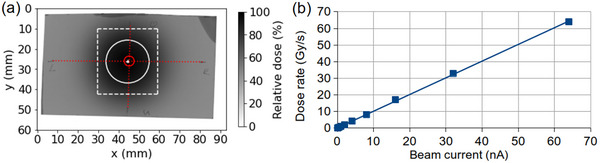
Position verification and dosimetry. (a) The correct positioning of the MRI scanner and the ionization chamber relative to the beam volume was verified using a sheet of radiochromic film attached to the proximal phantom surface. The white dotted rectangle denotes the area considered in the Gaussian fit used to determine the beam center and the 1 σ beam width marked by the white cross and circle, respectively. The red dotted lines represent the crosshair marked on the phantom surface, which guided the centered positioning of the ionization chamber on the container, while the red circle denotes the sensitive measurement volume of this chamber. (b) The results of the beam current to dose rate calibration were fit by a regression line of f(*x*) = 1.0038*x* + 0.1900 with R^2^ = 0.9996.

Dosimetric measurements were performed to verify the centered positioning of the MRI scanner, the phantoms and the ionization chamber relative to the beam and to acquire a reference depth‐dose distribution of the proton pencil beam as well as to establish a beam current setting to dose rate relation. A cylindrical, plane‐parallel ionization chamber with a sensitive volume of 0.02 cm^3^, 2.5 mm radius and 1 mm electrode distance (Advanced Markus Chamber Type 34045, PTW Freiburg GmbH, Freiburg, Germany) fastened to the proximal phantom surface at the position marked in green (Figure 2c) was used for the beam current to dose rate calibration performed using 207 MeV proton beams at beam currents ranging from 0.05 to 64 nA. Since recombination effects were negligible,^11^ the integral dose readings obtained could directly be interconverted into dose rates by mathematical division by the irradiation durations ranging from 2 to 10 s for the highest and lowest currents used, respectively. Radiochromic film (Gafchromic EBT3, Ashland, Wilmington, DE, USA) calibrated for clinical proton radiation fields at a few Gray dose level^11^ was used to verify the correct positioning of the scanner, the phantoms and the ionization chamber (batch number 04181701) as well as for depth‐dose profile measurement (batch number 11192002). Positioning verification was performed by irradiation of sheets of radiochromic film attached vertically to the proximal surface of the phantoms positioned at the MRI isocentre. The centered positioning of the scanner and the ionization chamber relative to the beam was verified by the evaluation of the beam position relative to phantom markings (Figure 2c) indicating the horizontal and vertical position of the MRI isocentre and the ionization chamber on the front surface of the main imaging and dosimetry phantom (Figure 3), which were transferred to the film prior to irradiation. The depth dose profile was measured using a sheet of radiochromic film screwed between two parallel PMMA plates angled by $1^{\circ }$ relative to the horizontal plane, which was irradiated in phantom position to an integral dose of 1.4 Gy using 207 MeV protons at a beam current of 32 nA. All films were scanned at a resolution of 300 dpi using 24‐bit color depth and evaluated using in‐house developed software.

### 2.6 Calculation of beam‐induced oxygen depletion and $T_{1}$ lengthening

Based on the measured dose rate, the corresponding oxygen depletion rate in the plateau region of the proton depth dose distribution was estimated from Figure 5b of a publication by Jansen and colleagues.^9^ The data underlying that study were previously obtained at the same proton research beamline as used for the present experiment, yet for a beam energy of 224 MeV instead of 207 MeV and for homogeneously irradiated small phantoms containing double deionized water. The total amounts of oxygen depletion were subsequently estimated for the respective integral doses applied over the irradiation durations used in the different experiments. In case the estimated amount of irradiation‐induced oxygen depletion, $\Delta c_{Ox,depletion}$, which was calculated independently from the initial oxygen concentration within a phantom, $c_{Ox,unirradiated}$, exceeded this initial oxygen concentration, full depletion was assumed to occur therein during the irradiation experiment by setting $\Delta c_{Ox,depletion}=-c_{Ox,unirradiated}$. The resulting oxygen concentration change‐dependent $T_{1}$ relaxation time after irradiation, $T_{1_{irradiated}}$, was obtained using

(5) $$T_{1_{irradiated}}=1/\left(1/T_{1_{unirradiated}}+r1_{Ox}\times \Delta c_{Ox,depletion}\right),$$

with the $T_{1}$ relaxation time of the unirradiated phantom estimated using Equation (3), $T_{1_{unirradiated}}$, and the relaxivity of oxygen, $r1_{Ox}$. The resulting $T_{1}$ lengthening was finally determined by subtraction of $T_{1_{unirradiated}}$ from $T_{1_{irradiated}}$.

### 2.7 Combined irradiation and imaging experiments

In all combined irradiation and imaging experiments, at least three images were acquired following a fixed timing (Figure 2d). A “before irradiation” image was acquired immediately before the irradiation was started. To allow for the equilibration of paramagnetic radical concentrations, the “during irradiation” acquisition was only started after a 30 s pre‐irradiation period,^8^ the irradiation being continued until the termination of this acquisition. The “after irradiation” acquisition was started immediately after the irradiation was terminated. All irradiations were performed using 207 MeV protons at 5 nA beam current with irradiation durations of 86 and 170 s for imaging at TRs of 2000 and 5000 ms, respectively.

#### 2.7.1 Spatial comparison of the beam‐induced MRI signature with a proton pencil beam dose distribution

To enable the spatial comparison of the proton beam‐induced MRI signature and a 207 MeV proton pencil beam dose distribution, an IR image with a TR of 5000 ms and a TI of 1460 ms was acquired of the “floral foam” phantom during simultaneous proton beam irradiation.

#### 2.7.2 Assessment of the temporal stability of the beam‐induced MRI signature

To assess the temporal stability of the observed beam‐induced signal change, IR images with a TR and TI of 5000 and 1460 ms, respectively, were acquired of the “floral foam” phantom at 0, 60, 120 and 170 min after irradiation.

#### 2.7.3 Assessment of the TR dependence of the beam‐induced MRI signature

To test the TR dependence of the observed beam‐induced contrast, two exemplary images with different TRs were acquired of the “agarose” phantom following the same 86 s irradiation. The first image was acquired 4 min post irradiation using a TR of 2000 ms and a TI of 755 ms, while the second was acquired with a TR and TI of 5000 and 1350 ms, respectively, at 27 min post irradiation.

#### 2.7.4 Assessment of the oxygen concentration and consumption rate dependence of the beam‐induced MRI signature

To investigate the dependence of the observed contrast on the initial oxygen concentration and the coumarin‐mediated modified oxygen consumption rate,^8^ IR images with a TR of 2000 ms and TIs of 755, 730 and 800 ms were acquired before, during and after proton beam irradiations of the “agarose”, “agarose + oxygen” and “agarose + coumarin” phantoms, respectively.

#### 2.7.5 Assessment of the feasibility of MRI‐based oxygen depletion imaging in tissue‐mimicking materials

To test the feasibility of oxygen depletion imaging in different tissue‐mimicking materials varying in initial oxygen concentration and homogeneity, IR images with a TR of 5000 ms and TIs of 1460, 240 and 110 ms were acquired before, during and after proton beam irradiations of the “floral foam”, “Lyoner sausage” and “pork liver” phantoms, respectively.

## 3 RESULTS

### 3.1 Determination of oxygen concentrations

The determined initial oxygen concentrations of the different phantoms are summarized in Table 1 and ranged from 1 μM in the “pork liver” to 342 μM in the “agarose + oxygen” phantom, compared to a reference value of 237 μM measured in distilled water.

**TABLE 1:** Temperatures, absorbed doses, inversion times, oxygen concentrations, $T_{1}$ relaxation times and the irradiation‐induced changes therein of all phantoms.

Phantom	$T{/}^{\circ }C$	D/Gy	TI/TR/ms	$c_{Ox,unirradiated}/\mu M$	$T_{1_{unirradiated}}$ / ms	$\Delta c_{Ox,depletion}/\mu M$	$\Delta T_{1}$/ms
Floral foam	28 ± 1	850 ± 43	1460/5000	197 ± 20	2660 ± 133	−170 ± 34	517 ± 346
Lyoner sausage	29 ± 1	850 ± 43	240/5000	23 ± 2	348 ± 17	−23 ± 5	1 ± 1
Pork liver	29 ± 1	850 ± 43	110/5000	1 ± 1	159 ± 8	−1 ± 1	0 ± 1
Agarose	28 ± 1	430 ± 22	755/2000	125 ± 13	2072 ± 104	−86 ± 17	142 ± 95
Agarose + coumarin	28 ± 1	430 ± 22	800/2000	137 ± 14	2232 ± 112	−137 ± 27	276 ± 185
Agarose + oxygen	28 ± 1	430 ± 22	730/2000	342 ± 76	1847 ± 92	−86 ± 17	112 ± 75

*Note*: The oxygen concentration measurements for all but the “agarose + oxygen” phantom were obtained using an optical probe. For the “agarose + oxygen” phantom, the mean oxygen concentration was estimated based on the experimentally determined TIs of all three agarose phantoms required to null the signal of the respective phantom at the given temperature and the oxygen concentrations measured in the “agarose” and “agarose + coumarin” phantoms. Moreover, no initial oxygen concentration‐dependent change in the oxygen consumption rate was assumed in the calculation of the total oxygen depletion in the “agarose + oxygen” phantom, although this has been observed previously ^8^ and may therefore be expected. For all measured or estimated values, the respective uncertainties are given. Symbols: T ‐ temperature, D ‐ absorbed dose, TI ‐ inversion time, $c_{Ox,unirradiated}$ ‐ oxygen concentration of the unirradiated phantom, $T_{1_{unirradiated}}$ ‐ $T_{1}$ relaxation time of the unirradiated phantom, $\Delta c_{Ox,depletion}$ ‐ irradiation‐induced oxygen depletion, $\Delta T_{1}$ ‐ change in $T_{1}$ relaxation time resulting from the oxygen depletion.

### 3.2 Position verification and dosimetry

**FIGURE 4:**
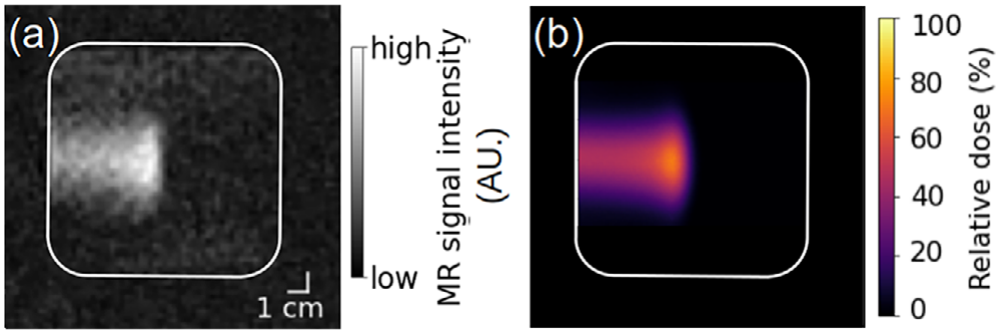
The IR image of the proton pencil beam stopping in the “floral foam” phantom shows good similarity to the deposited dose distribution. (a) Beam‐induced $T_{1}$ relaxation time contrast acquired during 850 Gy proton beam irradiation of the tap water‐soaked “floral foam” phantom. (b) Planar proton pencil beam dose distribution acquired during 1.4 Gy irradiation of radiochromic film. In both cases, the 207 MeV proton beams entered the phantoms from the left. The white contour represents the outer boundary of the phantom. IR, inversion recovery.

The proton beam was found to impinge centrally on the main phantom positioned at the MRI isocentre, confirming correct scanner and ionization chamber positioning with offsets of 0.8 and 0.3 mm in horizontal (*x*) and vertical (*y*) directions, respectively (Figure 3a). The dose rates measured for beam currents of 0.05 to 64 nA ranged from 0.1 to 63.9 Gy/s (Figure 3b). Linear regression yielded a dose rate of 5 Gy/s for 5 nA beam current irradiation, resulting in integral doses of 430 and 850 Gy in the plateau region of the depth dose profile (Figure 4b) for the 86 and 170 s irradiations performed in the experiments, respectively.

### 3.3 Calculation of beam‐induced oxygen depletion and $T_{1}$ lengthening

For the dose rate of 5 Gy/s, an oxygen depletion rate of −0.2 μM/Gy was read from plotted oxygen depletion rate measurements obtained in homogeneously irradiated double deionized water.^9^ This oxygen depletion rate was assumed to be valid in the plateau region of the proton dose distribution and for all phantom materials used in our study, resulting in maximum estimated depletions of oxygen of −86 and −170 μM for the integral doses of 430 and 850 Gy applied, respectively. Based on simulations,^8^ a doubling of the oxygen depletion rate was assumed for the “agarose + coumarin” phantom. Using the unit conversion factors provided in the supplement of the published empirical model, the relaxivity of oxygen was estimated to be 0.36/s/mM for a phantom temperature of $28^{\circ }C$ and a magnetic field strength of 0.32 T and assumed to be valid in all phantoms, although the model used is invalid in tissue.^13^ The initial $T_{1}$ relaxation time estimates for the individual phantoms ranged from 159 to 2660 ms for TIs ranging from 110 to 1460 ms. Considering the initial oxygen concentrations of the individual phantoms, the corresponding maximum changes in the oxygen concentrations and the resulting lengthening of the $T_{1}$ relaxation times amounted to −170 μM and 517 ms, respectively. A detailed overview of these values is provided in Table 1.

### 3.4 Combined irradiation and imaging experiments

#### 3.4.1 Spatial comparison of the beam‐induced MRI signature with a proton pencil beam dose distribution

A local magnitude signal hyperintensity was observed in the IR image acquired during simultaneous irradiation of the “floral foam” phantom (Figure 4a). This induced MRI signature resembled the corresponding proton pencil beam dose distribution in position and shape. Moreover, its local variation of signal intensity showed similarity with the local variation in relative dose within the beam region measured by radiochromic film dosimetry (Figure 4b).

#### 3.4.2 Assessment of the temporal stability of the beam‐induced MRI signature

**FIGURE 5:**
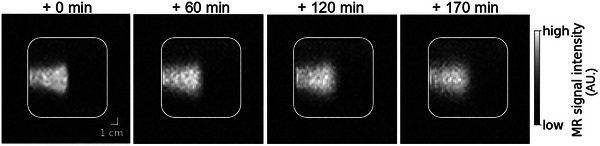
Proton beam‐induced $T_{1}$ relaxation time contrast persists over several hours. Time series of IR images acquired 0 to 170 min post irradiation of the “floral foam” phantom. IR, inversion recovery.

The observed local signal hyperintensity persisted over several hours. Over time, the contour of the MRI signature softened simultaneously with a gradual redistribution of the hyperintense signal within the phantom (Figure 5).

#### 3.4.3 Assessment of the TR dependence of the beam‐induced MRI signature

**FIGURE 6:**
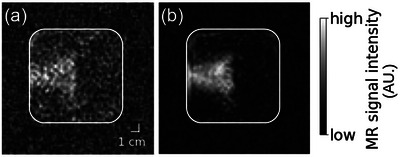
Beam‐induced $T_{1}$ relaxation time contrast depends on the TR of imaging. Exemplary IR images acquired of the “agarose” phantom (a) 4 min post irradiation using a TR of 2000 ms and (b) 27 min post irradiation using a TR of 5000 ms. IR, inversion recovery.

An increase in the TR from 2000 (Figure 6a) to 5000 ms (Figure 6b) enhanced the mean beam‐induced $T_{1}$ relaxation time contrast relative to the background signal by a factor of 3 as compared to the fourfold increase estimated using Equation (1).

#### 3.4.4 Assessment of the oxygen concentration and consumption rate dependence of the beam‐induced MRI signature

**FIGURE 7:**
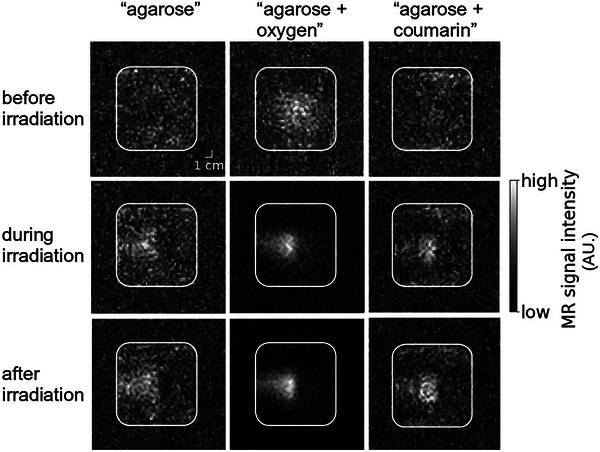
Beam‐induced $T_{1}$ relaxation time contrast depends on the initial oxygen concentration and the rate of oxygen consumption. IR images were acquired of the “agarose” phantom (first column), the oxygen concentration‐enhanced “agarose + oxygen” phantom (second column) and the oxygen consumption‐enhanced “agarose + coumarin” phantom (third column) before (first row), during (second row) and after irradiation (third row). All phantoms were irradiated using the same irradiation parameters. IR, inversion recovery.

While the beam‐induced hyperintense contrast was very weak in the beam visualization images acquired during and immediately after irradiation of the “agarose” phantom, it was strongly enhanced in the “agarose + oxygen” phantom, but only slightly enhanced in the “agarose + coumarin” phantom. The corresponding contrast enhancements relative to the background signal were 2.0, 3.3, and 2.3, respectively, for all three phantom images acquired immediately after irradiation (Figure 7).

#### 3.4.5 Assessment of the feasibility of MRI‐based oxygen depletion imaging in tissue‐mimicking materials

**FIGURE 8:**
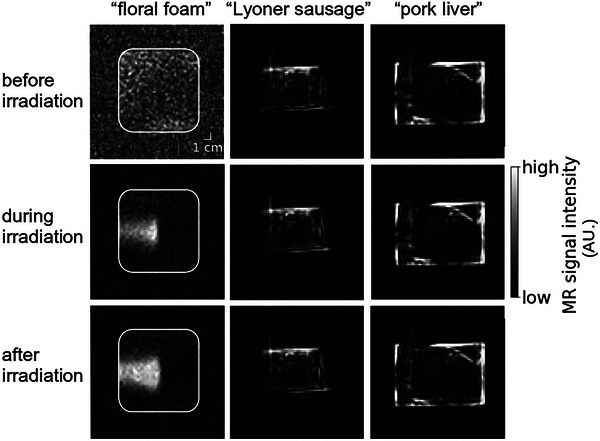
No proton irradiation‐induced contrast was observed in poorly oxygenated tissue‐mimicking phantoms. The initial oxygen concentration was the highest in the homogeneous “floral foam” phantom (first column) being 197 μM and decreased by an order of magnitude in the inhomogeneous “Lyoner sausage” (second column) and “pork liver” phantoms (third column) each. All proton beam irradiations were performed using the same irradiation parameters, the beam entering the phantoms from the left. Images were acquired before (first row), during (second row) and immediately after irradiation (third row).

While clear beam‐induced hyperintense $T_{1}$ relaxation time contrast was observed in the well oxygenated “floral foam” phantom during and, further intensified, after irradiation, no such beam‐induced contrast was visible in the poorly oxygenated tissue‐mimicking “Lyoner sausage” and “pork liver” phantoms at any timepoint. While the phantom background signal could be nulled in the homogeneous “floral foam” phantom, no complete background signal nulling was achieved in the other two more inhomogeneous phantoms (Figure 8).

## 4 DISCUSSION

For the first time, a flow‐independent contrast mechanism for MRI‐based proton beam visualization in real‐time was identified by generating experimental evidence of the feasibility of the MRI‐based detection of proton beam‐induced local oxygen depletion in convection‐restricted aqueous phantoms.

The hypothesis that the proton‐beam induced radiochemical depletion of paramagnetic oxygen may result in MRI‐detectable $T_{1}$ relaxation time changes was mainly based on a similar, but not convection‐controlled study,^8^ in which photon beam‐induced negative MR image contrast ascribed to oxygen depletion was first observed using an MR‐linac. In the radiochemistry simulation additionally performed in that study, it was moreover demonstrated that the concomitant change in the paramagnetic free radical concentrations did not strongly influence the observed image contrast. Based on literature, a high degree of similarity in the radiochemistry of low linear energy transfer photon and proton irradiation can be expected.^9^, ^14^ Consequently, guided by previous studies,^8^, ^9^ the high dose and dose rate proton beam irradiations in the present study were designed to result in MRI‐detectable levels of oxygen depletion‐induced $T_{1}$ relaxation time changes. Simultaneously, the full depletion of oxygen was avoided to still enable contrast verification by chemically enhanced oxygen consumption rates.

The $T_{1}$ relaxation time changes in the order of $10^{2}$ ms for well oxygenated phantoms were estimated using a framework previously published and experimentally verified.^8^ The estimates of the overall irradiation‐induced oxygen depletion underlying these estimated $T_{1}$ relaxation time changes were estimated to suffer from about 20% uncertainty. This uncertainty resulted from the estimated 15% uncertainty in the oxygen depletion rate, which was expected to be dominated by imprecise reading and the differences in the initial oxygen concentrations used^10^ rather than by the comparatively small uncertainties in the dose rate measurement and the small deviations in the proton beam energies.^15^ Another 5% uncertainty originated from the absorbed dose measurement. Combined with the uncertainty in the relaxivity value of oxygen of 15%,^13^ these uncertainties translated into an overall uncertainty in the $T_{1}$ relaxation time change values of 67%. Nevertheless, the estimated $T_{1}$ relaxation time changes were likely considered sufficiently large for the MRI‐based detection in our study, considering the detection thresholds for $T_{1}$ relaxation time changes in the order of $10^{2}$  ms determined in reference experiments for IR imaging at TRs of 2000 and 5000 ms, respectively (see Supplementary Information Figure and Table S1).

The oxygen depletion hypothesis for MRI‐based proton beam visualization was assessed in a series of four experiments testing the expected dose dependence, temporal stability, TR dependence as well as the dependence on the initial oxygen concentration and oxygen consumption rate. The first of these beam visualization experiments comparing the proton beam‐induced MRI magnitude signature with a proton pencil beam dose distribution qualitatively captured the expected linear dose dependence of oxygen depletion^9^, ^10^ in combination with an approximately linear oxygen depletion‐related MR signal change as predicted by theory. Moreover, the spatial similarity of the MRI signature and the proton dose distribution hints at a possible applicability of this method for geometric QA tasks as demonstrated previously for convection‐based proton beam visualization in liquids.^4^, ^7^ In the second experiment, the beam‐induced signature was found to persist for several hours, which was in line with previous measurement^9^ and simulation^8^ results reporting stable lowered oxygen concentrations for tens of minutes after irradiation. The gradual redistribution of signal over time may reflect a slow diffusion effect.^17^ The third experiment revealed the susceptibility of the observed contrast to changes in TR with a measured threefold intensification of contrast at longer TR. This observation is in line with the fourfold increase in contrast predicted using Equation (1), which, however, neglects the influence of noise. The experimental verification of this predicted increase in the absolute signal of spins with $T_{1}$ relaxation times which differ from that of the signal‐nulled background confirms that the observed contrast was truly evoked by an irradiation‐induced change in the $T_{1}$ relaxation time of water. In the fourth experiment, the beam‐induced contrast was found to markedly intensify with increased initial oxygen concentration. Yet, influenced by the initial $T_{1}$ relaxation time values and the assumed oxygen concentration‐independent oxygen depletion rate, theory predicted the change in $T_{1}$ relaxation time, which is approximately linear to a change in magnitude signal, to be the smallest in the “agarose + oxygen” phantom relative to in the other agarose‐based phantoms. Unfortunately, studies investigating the dependence of oxygen depletion rates on the initial oxygen concentrations do not cover above‐atmospheric oxygen concentrations for conditions matching our experiment.^10^, ^18^ Therefore, it can only be hypothesized that either the oxygen depletion rates are strongly enhanced in phantoms with initial oxygen concentrations increased above atmospheric levels or that oxygen is already fully depleted by the irradiation in the other agarose‐based phantoms with lower initial oxygen concentrations. The latter explanation seems possible in our own study but very unlikely in the comparatively low dose study on oxygen depletion imaging conducted on an MR‐linac, which also reported higher irradiation‐induced contrast in a phantom with above‐atmospheric initial oxygen concentration.^8^ Unfortunately, no independent oxygen concentration measurements were performed in that study. Moreover, because of the coumarin‐related twofold increase in the oxygen consumption rate,^8^ a 94% increase in oxygen depletion‐related $T_{1}$ relaxation time lengthening was estimated for the “agarose + coumarin” relative to the “agarose” phantom. Yet, the small 5% increase in contrast observed could further support the hypothesis of almost complete oxygen depletion induced in these two phantoms by irradiation alone.

The main limitations of the current methodology are the poor signal‐to‐noise ratio (SNR) in combination with varying noise levels and the limited temporal resolution of imaging. These limitations contribute to the currently achievable low sensitivity of imaging to oxygen depletion, result in unfavorable averaging of the time‐varying concentration changes and may therefore affect the overall reliability and precision of the results. Nevertheless, in summary, the experimental findings strongly support the hypothesis that the observed MR signal changes were evoked by proton beam irradiation‐induced oxygen depletion.

This newly established contrast mechanism for MRI‐based proton beam visualization may also well explain the hypointense time‐of‐flight angiography magnitude signal change previously observed in irradiated water under convection‐restricted conditions.^7^ Because this previous experiment was conducted at even higher dose levels, pronounced irradiation‐induced oxygen depletion could be expected, which, using the present framework for the calculation of the concomitant $T_{1}$ relaxation time changes, could be expected to translate into a just visible signal change.

The last experiment of this study was conducted to further experimentally assess the method's potential for MRI‐based beam detection in tissue, a subject already investigated.^8^ Using different initial oxygenation levels down to the physioxic (14–149 μM) and hypoxic (<14 μM) range^19^ in the tissue‐mimicking “Lyoner sausage” and “pork liver” phantoms, respectively, no oxygen depletion‐induced contrast could be generated in the present study for doses of 850 Gy. Most likely, the low initial oxygen concentrations limited the achievable levels of oxygen depletion, resulting in $T_{1}$ relaxation time changes below the detection limit. Moreover, the smaller initial $T_{1}$ relaxation times of tissue compared to fluids further decreased the $T_{1}$ relaxation time differences resulting from a given change in oxygen concentration. Additionally, the detection sensitivity was compromised by the poorer background signal nulling achievable in these more inhomogeneous phantoms which might be mitigable by working at higher resolution in combination with the acquisition of a map of the initial $T_{1}$ relaxation times to inform a subsequent signal correction step in data processing.

A possible application of MRI‐based oxygen depletion imaging in in vivo FLASH treatment with high fraction doses applied at ultra‐high dose rates has been suggested, but was simultaneously concluded to pose high, currently unmet SNR and temporal resolution requirements.^8^ Considering the results of our last experiment, the shorter $T_{1}$ relaxation times in tissue, as well as their inhomogeneity may well be other decisive, unmodifiable factors exacerbating oxygen depletion‐based beam detection in tissue in the future. To assess whether MRI can simultaneously achieve both, the necessary temporal and $T_{1}$ relaxation time resolution required for the detection of small, transient oxygen depletion‐induced $T_{1}$ relaxation times changes in tissue, future studies will be required, which use fast imaging and accurately model reoxygenation processes.

## 5 CONCLUSION

By demonstrating the feasibility of the MRI‐based detection of proton beam‐induced local oxygen depletion in convection‐restricted aqueous phantoms, a first flow‐independent contrast mechanism for MRI‐based proton beam visualization in real‐time has been identified. Besides a possible application of this method in phantom‐based QA of MR‐integrated proton therapy systems, its eligibility for in vivo treatment verification in FLASH radiotherapy should be further assessed. To achieve applicability in realistic clinical scenarios, the current limitations that lie in poor SNR and temporal resolution as well as in a lack of mitigation strategies for the sensitivity loss due to baseline $T_{1}$ relaxation time inhomogeneity of tissue must be addressed in future studies.

## CONFLICT OF INTEREST STATEMENT

The authors have no relevant conflicts of interest to disclose.

## Supporting information

Supporting Information

## References

[1] Newhauser WD , Zhang R . The physics of proton therapy. Phys Med Biol. 2015;60(8):R155. 25803097 10.1088/0031-9155/60/8/R155 PMC4407514

[2] Hoffmann A , Oborn B , Moteabbed M , et al. MR‐guided proton therapy: a review and a preview. Radiat Oncol. 2020;15:1‐13. 10.1186/s13014-020-01571-x PMC7260752 32471500

[3] Moteabbed M , Smeets J , Hong TS , et al. Toward MR‐integrated proton therapy: modeling the potential benefits for liver tumors. Phys Med Biol. 2021;66(19):195004. 10.1088/1361-6560/ac1ef2 34407528

[4] Schellhammer S . Technical Feasibility of MR‐Integrated Proton Therapy: Beam Deflection and Image Quality. PhD thesis. Technische Universität Dresden; 2019. https://nbn‐resolving.org/urn:nbn:de:bsz:14‐qucosa2‐341326

[5] Gantz S , Karsch L , Pawelke J , et al. Direct visualization of proton beam irradiation effects in liquids by MRI. Proc Natl Acad Sci USA. 2023;120(23):e2301160120. 37252953 10.1073/pnas.2301160120 PMC10265969

[6] Schieferecke J , Gantz S , Hoffmann A , Pawelke J . Investigation of contrast mechanisms for MRI phase signal‐based proton beam visualization in water phantoms. Magn Reson Med. 2023;90(5):1776‐1788. 37345700 10.1002/mrm.29752

[7] Schieferecke J , Gantz S , Karsch L , Pawelke J , Hoffmann AL . MRI magnitude signal‐based proton beam visualisation in water phantoms reflects composite effects of beam‐induced buoyant convection and radiation chemistry. Phys Med Biol. 2023;68(18):185002. 10.1088/1361-6560/acf2e0 37607554

[8] Wancura J , Egan J , Sajo E , Sudhyadhom A . MRI of radiation chemistry: first images and investigation of potential mechanisms. Med Phys. 2023;50(1):495‐505. 36201151 10.1002/mp.16011 PMC9930196

[9] Jansen J , Knoll J , Beyreuther E , et al. Does FLASH deplete oxygen? Experimental evaluation for photons, protons, and carbon ions. Med Phys. 2021;48(7):3982‐3990. 33948958 10.1002/mp.14917

[10] El Khatib M , Van Slyke AL , Velalopoulou A , et al. Ultrafast tracking of oxygen dynamics during proton FLASH. Int J Radiat Oncol Biol Phys. 2022;113(3):624‐634. 35314293 10.1016/j.ijrobp.2022.03.016 PMC9250619

[11] Beyreuther E , Brand M , Hans S , et al. Feasibility of proton FLASH effect tested by zebrafish embryo irradiation. Radiother Oncol. 2019;139:46‐50. 31266652 10.1016/j.radonc.2019.06.024

[12] Bernstein MA , King KF , Zhou XJ . Handbook of MRI Pulse Sequences. Elsevier; 2004.

[13] Bluemke E , Stride E , Bulte DP . A simplified empirical model to estimate oxygen relaxivity at different magnetic fields. NMR Biomed. 2022;35(2):e4625. 34599536 10.1002/nbm.4625 PMC11475509

[14] Burns WG , Sims HE . Effect of radiation type in water radiolysis. J Chem Soc, Faraday Trans 1. 1981;77(11):2803‐2813.

[15] Sanchez‐Parcerisa D , Cortés‐Giraldo M , Dolney D , et al. Analytical calculation of proton linear energy transfer in voxelized geometries including secondary protons. Phys Med Biol. 2016;61(4):1705. 26840945 10.1088/0031-9155/61/4/1705

[16] Cao X , Zhang R , Esipova TV , et al. Quantification of oxygen depletion during FLASH irradiation in vitro and in vivo. Int J Radiat Oncol Biol Phys. 2021;111(1):240‐248. 33845146 10.1016/j.ijrobp.2021.03.056 PMC8338745

[17] Rothwell BC , Kirkby N , Merchant MJ , et al. Determining the parameter space for effective oxygen depletion for FLASH radiation therapy. Phys Med Biol. 2021;66(5):055020. 10.1088/1361-6560/abe2ea PMC8208623 33535191

[18] Boscolo D , Krämer M , Fuss MC , Durante M , Scifoni E . Impact of target oxygenation on the chemical track evolution of ion and electron radiation. Int J Mol Sci. 2020;21(2):424. 31936545 10.3390/ijms21020424 PMC7014692

[19] Carreau A , Hafny‐Rahbi BE , Matejuk A , Grillon C , Kieda C . Why is the partial oxygen pressure of human tissues a crucial parameter? Small molecules and hypoxia. J Cell Mol Med. 2011;15(6):1239‐1253. 21251211 10.1111/j.1582-4934.2011.01258.x PMC4373326

