# Supplementary material for: MR imaging of proton beam‐induced oxygen depletion
Source: Med Phys. 2025 Jan 28;52(4):2454–64. doi: 10.1002/mp.17622 (PMC11972052; doi:10.1002/mp.17622)
Supplement: Supplementary file 1 — Supporting Information [file MP-52-2454-s001.pdf]

Supplementary Information

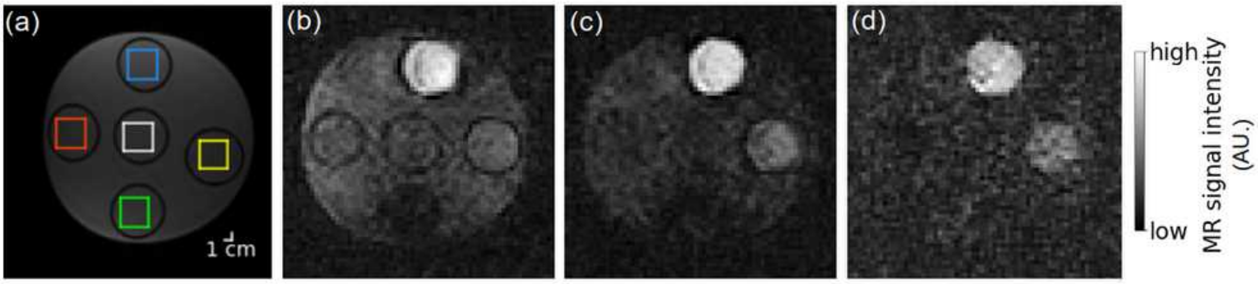

Figure S1: T_1 relaxation time difference detection sensitivity of IR imaging using the MrJ3300 scanner. (a) T_1 relaxation time phantom comprising compartments of 0.30 M (blue), 0.15 M (yellow), 0.07 M (green) and 0.03 M (red) sucrose solution and the solvent distilled water (white), which also surrounds all inset vials. (b) IR contrast at TR = 5000 ms and TI = 1515 ms. (c) IR contrast at TR = 5000 ms and TI = 1528 ms. (d) IR contrast at TR = 2000 ms and TI = 813 ms. While each of the three highest sucrose concentrations can individually be discriminated from distilled water based on their individual absolute T_1 relaxation time differences relative to water at a TR of 5000 ms under conditions where either the water or the respective sucrose solution signal is nulled by choice of a nulling TI, this is only true for the two highest sucrose concentrations at a TR of 2000 ms.

Table S1: T_1 relaxation time measurements of phantoms with different sucrose concentrations. For the five different sucrose concentrations (columns) used in the phantom shown in figure S1, T_1 relaxation time estimates were obtained in four different quantitative experiments (lines) using either the multiple TI inversion recovery method[A] (experiments 1 and 2) or the signal-nulling TI-based method (experiments 3 and 4), which was also used in the oxygen depletion beam visualisation experiments. To obtain the individual T_1 relaxation time estimates provided in the table using the multiple TI inversion recovery method, three IR images each were acquired at TIs of 80, 640 and 1280 ms of every phantom in both experiments using the MrJ3300 scanner and the signal intensities averaged before being fed into the calculations to increase the robustness of the T_1 estimates obtained. To obtain the mean T_1 estimates provided in line 6, the T_1 estimates obtained in the individual experiments were averaged for further increased robustness. Experiment (Exp.) 1: T_1 relaxation time measurements were performed in individual sucrose phantoms measured protracted over time, but at the same position within the field of view using the cervical spine receiver coil and a TR of 15000 ms; Exp. 2: Simultaneous measurement of the T_1 relaxation times of all sucrose concentrations in the phantom presented in figure S1 using the knee receiver coil, the phantom being imaged in two different 180° rotated orientations at a TR of 15000 ms; Exp. 3 and 4: T_1 relaxation time estimation based on experimentally determined TIs required to null the signal of the individual sucrose concentrations with TR = 2000 and 5000 ms, respectively, using the knee coil and phantom shown in figure S1.

Sucrose concentration / M	0	0.03	0.07	0.15	0.30
Exp. 1: T_1 estimate / ms	2734	2796	2652	2742	2200
Exp. 2: T_1 estimate / ms	2938	2888	2705	2424	2928
Exp. 3: T_1 estimate / ms	2849	2796	2762	2485	2232
Exp. 4: T_1 estimate / ms	2902	2910	2891	2711	2469
Mean T_1 estimate ± standard deviation / ms	2856 ± 89	2848 ± 60	2753 ± 103	2591 ± 160	2457 ± 336
Absolute mean T_1 difference relative to 0 M sucrose / ms	-	8 ± 149	103 ± 192	265 ± 249	399 ± 425

Interpretation:
The absolute mean T_1 relaxation time differences within the vials containing different sucrose concentrations relative to the reference 0 M sucrose sample were used to estimate the imaging parameter-dependent T_1 relaxation time difference sensitivity of the IR pulse sequence. Because the 0.07 M sucrose sample was the lowest concentration distinguishable from 0 M sucrose using the IR pulse sequence with the longer TR setting of 5000 ms (Figure S1b), the detection sensitivity for T_1 relaxation time differences was assumed to lie around 103 ± 192 ms. In the same way, a lower sensitivity limit of approximately 265 ± 249 ms was assumed for a TR of 2000 ms (Figure S1d). Because of the large uncertainties associated with these measurements, the determined sensitivity limits can only be interpreted as rough order-of-magnitude estimates.

References

[A] Brown, Robert W., et al. *Magnetic resonance imaging: physical principles and sequence design*. John Wiley & Sons, 2014.
